# Constructing a neutrophil extracellular trap model based on machine learning to predict clinical outcomes and immune therapy responses in oral squamous cell carcinoma

**DOI:** 10.3389/fgene.2025.1616868

**Published:** 2025-09-08

**Authors:** Jian Wang, Zhenzhen Li, Zhiwei Li, Zijing Yu, Wenpin Xu

**Affiliations:** ^1^ Department of Stomatology, Taihe Hospital, Hubei University of Medicine, Shiyan, Hubei, China; ^2^ Department of Cardiac Function, Cardiovascular Diagnosis and Treatment Center, Taihe Hospital, Hubei University of Medicine, Shiyan, Hubei, China

**Keywords:** neutrophil extracellular traps, oral squamous cell carcinoma, LINC00937, prognostic model, non-negative matrix factorization, machine learning

## Abstract

**Background:**

Neutrophil extracellular traps (NETs) represent a novel form of inflammatory cell death in neutrophils. Recent studies suggest that NETs can promote cancer progression and metastasis through various mechanisms. This study focuses on identifying prognostic NETs signatures and therapeutic targets for oral squamous cell carcinoma (OSCC).

**Materials and Methods:**

We performed non-negative matrix factorization (NMF) analysis on 89 previously reported NET-related genes within the TCGA cohort. Subsequent analysis of subtype feature genes was conducted using the weighted gene co-expression network analysis (WGCNA). Six machine learning algorithms were employed for model training, with the best model selected based on 1-year, 3-year, and 5-year AUC values. A NETs signature was developed to predict overall survival in OSCC patients. Multi-omics validation was carried out, and stable knockout OSCC cell lines for key genes were established to assess the biological functions of LINC00937 *in vitro*.

**Results:**

Five NETs-related clusters were identified in OSCC patients, with the C5 subtype showing the most favorable prognosis. The WGCNA network revealed 443 characteristic genes. The Enet algorithm exhibited optimal performance in providing a predictive NETs signature. Multi-omics analysis indicated that NETs signaling is linked to an immunosuppressive microenvironment and can predict the efficacy of immunotherapy. *In vitro* experiments confirmed that knocking down LINC00937 led to inhibited tumor growth.

**Conclusion:**

This study highlights the emerging role of NETs in OSCC, presenting a prognostic NETs feature and identifying LINC00937 as a significant factor in OSCC. These findings contribute to risk stratification and the discovery of new therapeutic targets for OSCC patients.

## Introduction

Clinically, oral squamous cell carcinoma (OSCC) is the most common malignant tumor among head and neck cancers, with high incidence and mortality (Yoshimura et al., 2021). The primary treatment modalities for OSCC include surgery, radiotherapy, and chemotherapy (Yoshida et al., 2020). However, the 5-year overall survival (OS) for OSCC has not significantly improved, remaining at approximately 45%–50% (Lu et al., 2023; Wu et al., 2022). Due to the unique location of OSCC, issues such as recurrence, metastasis, and chemotherapeutic drug resistance continue to pose significant challenges. Consequently, the identification of new prognostic biomarkers and valuable therapeutic targets for OSCC has been a primary focus of research. The immune cells in OSCC and the immune responses within the tumor microenvironment (TME) have garnered significant attention from researchers (Xiong et al., 2022). As critical members of the TME, neutrophils are functionally closely associated with the progression of OSCC (Xu et al., 2021; Bai et al., 2022).

Neutrophils within the TME are considered crucial pathogenic factors contributing to tumor progression (Cui et al., 2021; Quail et al., 2022). In particular, neutrophil extracellular traps (NETs) produced by neutrophils have been identified as a novel pro-tumorigenic mechanism in various cancers (Branzk et al., 2014). NETs are web-like structures composed of DNA, histones, and granules released from activated neutrophils (Fuchs et al., 2007). Previous studies have shown that NETs levels in the peripheral blood of healthy individuals are lower compared to those in cancer patients (Rayes et al., 2019; Jin et al., 2019). Additionally, *in vitro* studies have demonstrated that co-culturing neutrophils with breast cancer and lung cancer tumor cells can induce NETs formation (Masucci et al., 2020; Wu et al., 2020; Park et al., 2016).

Recent studies have indicated that NETs play a tumor-suppressive role within the TME (Han et al., 2024). On one hand, NETs can repel cytotoxic cells and attract immunosuppressive cells. On the other hand, NETs can serve as a physical barrier, preventing antibody-dependent cellular cytotoxicity (ADCC) (Teijeira et al., 2021). Notably, NETs can express PD-L1, leading to the exhaustion of CD8^+^ T cells within the TME. Anti-NET therapies might enhance immune cell-mediated tumor cell killing and synergize with immune checkpoint inhibitors (ICIs) (Kaltenmeier et al., 2021; Wang et al., 2021). Therefore, combining anti-NET treatments with ICIs could potentially reduce the incidence of ICI resistance and offer a new therapeutic strategy for cancer treatment.

Currently, a novel anticancer strategy involves inhibiting the formation of NETs or promoting the degradation of NETs within tumors (Papayannopoulos et al., 2010). Unfortunately, drugs targeting NETs have not yet been developed, thus failing to improve the prognosis of cancer patients (Lewis et al., 2015). Targeted therapies that modulate neutrophil recruitment and NET formation in preclinical models suggest new therapeutic strategies, specifically targeting the upstream mediators of NET formation in tumors (Tang et al., 2020). Therefore, exploring the upstream mediators of NETs is becoming an essential component of targeted therapy.

Given the immunosuppressive functions of NETs within the tumor microenvironment and their potential role in promoting resistance to immune checkpoint inhibitors, targeting NETs has emerged as a promising therapeutic strategy in several cancers. However, in OSCC, the clinical relevance and therapeutic implications of NETs remain poorly understood. In this study, we developed a prognostic NETs biomarker for patients with OSCC using machine learning approaches. Among the genes comprising this NETs signature, LINC00937 was identified as a key feature gene due to its prognostic relevance and elevated expression in tumor tissues. Functional experiments demonstrated that LINC00937 promotes proliferation, migration, and invasion of OSCC cells, suggesting a strong oncogenic role. Although direct evidence linking LINC00937 to NET formation is lacking, we speculate that it may be involved in NET-associated regulatory processes. Overall, our findings not only provide a robust prognostic tool but also highlight LINC00937 as a potential therapeutic target, contributing to a deeper understanding of NET biology in OSCC and informing future immunotherapy strategies.

## Materials and methods

### Data download and processing

In this study, all data were obtained from publicly available online sources. Gene expression data and clinical data for OSCC were downloaded from the TCGA database (https://portal.gdc.cancer.gov/), including 314 RNA-seq datasets and 314 clinical datasets. Additionally, OSCC gene expression data were downloaded from the GEO database (https://www.ncbi.nlm.nih.gov/geo/), including GSE31056 (n = 96) (Reis et al., 2011), GSE41613 (n = 97) (Lohavani et al., 2013), GSE42743 (n = 103) (Zhao et al., 2022), and GSE85446 (n = 66) (van Hooff et al., 2012).

The data obtained from GEO was corrected and normalized using the R package “limma” (v 3.50.1), followed by log2 transformation. Batch effects between datasets were then removed using the R package “ComBat”. For the data obtained from TCGA, gene ID annotation was performed using the Ensemble database. In cases where genes had duplicate names, only the gene with the highest expression level was retained. RNA-seq expression data downloaded from TCGA was provided in transcripts per million (TPM), and gene expression levels were normalized using log2 (TPM+1).

### Non negative matrix factorization clustering based on NETs related genes

In previous studies, a total of 89 NETs-related genes were identified (Wang et al., 2020; Zhang et al., 2022). Non-negative matrix factorization (NMF) was employed to perform clustering analysis on the TCGA cohort based on the expression levels of these NETs-related genes. Since the number of clusters (k) in NMF must be manually specified, we tested a range of k-values from 3 to 8. This range was selected based on prior studies involving molecular subtype identification in cancer, where 3 to 8 subtypes are commonly observed and considered biologically meaningful. For each k-value within this range, NMF was run with 50 iterations to ensure stability of clustering results. We then evaluated clustering quality using the product of the cophenetic correlation coefficient and the dispersion coefficient—two commonly used metrics reflecting cluster robustness and separation. The k-value that yielded the highest product of these two metrics was selected as the optimal number of clusters. Finally, NMF was performed with 500 iterations using the optimal k to identify stable NETs-related subtypes in OSCC. All procedures were conducted using the R package “nmf”.

### Enrichment analysis and single sample gene set enrichment analysis

The R package “clusterProfiler” (v 4.2.2) was used for performing Gene Ontology (GO) enrichment analysis and KEGG pathway enrichment analysis to predict the biological functions of genes. Enrichment analysis results with a p-value <0.05 were considered statistically significant.

Hallmark and immune cell infiltration gene sets were collected from the MSigDB database (https://www.gsea-msigdb.org/gsea/index.jsp). Gene Set Variation Analysis (GSVA) using the “gsva” function in the R package “GSVA” (v 1.44.3) was employed for single-sample gene set enrichment analysis (ssGSEA) to evaluate the standardized enrichment scores (NESs) of gene sets in each patient. The R package “estimate” was utilized to compute StromalScore, ImmuneScore, ESTIMATEScore, and TumorPurity for each subtype.

### Construction of WGCNA network

Weighted Gene Co-expression Network Analysis (WGCNA) is a systems biology approach that identifies modules of highly correlated genes based on their relationships with gene sets and phenotypes, aiming to discover candidate biomarker genes and potential therapeutic targets. In this study, we used the gene expression profiles of OSCC from TCGA to construct a WGCNA network. First, samples and genes were filtered, and then the R package “WGCNA” (v 1.71) was used to calculate the Pearson correlation between all pairs of genes in the selected samples, constructing an adjacency matrix. To ensure a scale-free network, we chose a soft-thresholding power β = 8 (R^2 = 0.90). Next, to further identify functional modules in the weighted gene co-expression network, we calculated the Topological Overlap Measure (TOM) based on the adjacency matrix. Using the TOM values, we employed a dynamic tree-cutting method to establish gene modules and selected module eigengenes (MEs). MEs are considered representative of the gene expression profiles within each module.

### Machine learning framework establishes prognostic NETs signature

To establish a robust NETs signature, we employed six machine learning algorithms on the TCGA dataset: CoxBoost, Elastic Net (Enet), Lasso, Random Forest (RF), Ridge Regression, and Support Vector Machine (SVM). Patients were randomly split into training (70%) and validation (30%) sets. Model performance was evaluated using 10-fold cross-validation to compute and compare the AUC values for 1-, 3-, and 5-year survival predictions. This approach was used to select the best NETs signature.

### Construction of predictive nomogram model

After selecting the optimal NETs signature, the Normalized Enrichment Scores (NESs) of the signature were calculated using ssGSEA. These NESs, along with other clinical features, were used to build a nomogram model to predict 1-, 3-, and 5-year overall survival rates for patients with OSCC. Calibration curves were established to assess the consistency between predicted and actual survival. The “rms” R package was used to construct the nomogram model.

### NETs signature annotates tumor microenvironment, signaling pathways, and immune-related features

We collected 5 classes of immune regulatory factors, including antigen presentation, immune inhibition, immune activation, chemokines, and receptors. Signal pathways related to targeted therapy and immunotherapy were collected from the MSigDB database, and NESs were calculated using ssGSEA. Five immune deconvolution methods were used to assess immune cell infiltration abundance in patients with OSCC, including quantiseq, CIBERSORT, MCPcounter, Xcell, and EPIC. The Tumor Immune Phenotype (TIP, http://biocchrbmu.edu.cn) was used to evaluate anticancer immune activity (Xu L. et al., 2018). The TIDE (http://tide.dfci.harvard.edu/) database was used for immune-related feature analysis (Avila Cobos et al., 2020).

### Patients and specimens

Ten pairs of OSCC patients with established OSCC diagnosis were collected by the Department of Stomatology Taihe Hospital, Hubei University of Medicine. In this study, none of the patients was treated with neoadjuvant therapy. Their OSCC samples and matched non-carcinoma tissue samples were first formalin-fixed, paraffin-embedded utilized for testing the expression of LINC00937. The Ethical Committees of the Taihe Hospital affiliated to Hubei University of Medicine permitted our study. Each tissue was provided the informed consent before participation.

### Fluorescence *in situ* hybridization (FISH)

Fluorescence *in situ* hybridization (FISH) was used to test the expression of LINC00937 by a Probe Mix kit (Servicebio, Wuhan, China). OSCC tissue and normal tissue were firstly fixed in 4% paraformaldehyde (Solarbio, China) for 10 min. The protocol was used previously. Then, the pre-hybridization pad was discarded, and 150 μL of the hybridization buffer was added with the lncRNA FISH Probe Mix. Hybridization was done at 37 °C overnight, followed by washing with different buffers at 42 °C. Nuclei were treatment with DAPI (Servicebio, Wuhan, China) and images collected by inverted fluorescence microscopy (Phenix, Jiangxi, China) in five random areas.

### Cell culture and siRNA transfection

CAL-27, were obtained from TongPai Biotechnology Co., LTD. (Shang Hai, China). OSCC cells were grown in DMEM (Gibco, United States) with 10% fetal bovine serum (Gibco, Australia) at 37 °C with a 5% CO2 incubator. LINC00937 siRNAs were obtained from RiboBIO (Guangzhou, China). siRNA sequences were:

si-h-LINC00937_001: 5-GAG​GAA​TAA​CTT​CAC​TCT​T-3;
si-h-LINC00937_002: 5-GTA​TAA​ATT​GAG​CTG​ACT-3;
si-h-LINC00937_003: 5-GAG​CTG​ACT​GCA​AGG​TAC​T-3;

The LINC00937-siRNA targeting si-LINC00937 or negative control si-NC by RiboFECTTM CP (RiboBIO, Guangzhou, China). Control siRNAs were standard as the negative control.

### RNA isolation and quantitative real-time qPCR

Total RNA was harvested and used for cDNA synthesis by Trizol reagent (Thermo Fisher Scientific, USA) and SuperScript II first-strand cDNA synthesis kit (Thermo Fisher Scientific, USA). RT-qPCR was detected by SYBR Premix Ex Taq (TaKaRa, China) based on the control. The 2^−ΔΔCT^ approach was used to determine expression levels relative to those of GAPDH. The primer sequences were as follow:

LINC00937:
Forward: 5′-CGG​GTC​CTT​CCT​CTT​CCC​CA-3’;
Reverse: 5′-CGC​AGC​CTC​TTC​TCT​TCG​GG-3′
GAPDH:
Forward: 5′-GAA​GGT​GAA​GGT​CGG​AGT​C-3′;
Reverse: 5′-GAA​GAT​GGT​GAT​GGG​ATT​TC-3′

### Cell growth and proliferation

CCK-8 (Cell Counting Kit 8, Servicebio, Wuhan, China) and colony formation analyses were employed to test the cell growth. For the CCK-8 comments, OSCC cells were first inoculated in 96-well plates with cell numbers of 6,000/well. The CCK-8 reagent was added to every well at varying time points (0, 24, 48, and 72 h), after which cells were stored at 37 °C for 2–4 h. All the cells were tested at the optical density (OD) of 450 nm by a microplate reader (Thermo Fisher Scientific, USA).

For colony formation assays, cells after treatment as before. After 14 days treatment cells crucial with 1% crystal violet Dissolve in methanol for 15–30 min.

### Trans-well assay

8-µm pore size chambers employed cell migration tests without the Matrigel gel. Cells with the number at 50,000 were inoculated into the upper section with a none-serum media. At the lower well stage, 20% FBS-supplemented medium was added. After 1 day of incubation, all cells in the wells were stained, after which optical microscopy (Phenix, Jiangxi, China) was performed for observation. Five random areas were collected for each sample. Chambers also performed cell invasion assays with Matrigel gel. Other procedures were performed as earlier described.

### Statistical analysis

We used the surv_cutpoint function from the R package “survminer” (v 0.4.9) to calculate the optimal cutoff values and stratify patients into groups. Kaplan-Meier survival curves were plotted using the R packages “survminer” and “survival” (v 3.3-1) to compare the differences in survival between different patient groups, assessed by the two-sided log-rank test. The R package “pROC” (v 1.18.0) was used for ROC curve analysis. Data visualization was conducted using the R package “ggplot2” (v 3.3.5). For continuous variable comparisons between two groups, we used the Wilcoxon rank-sum test for unpaired data and paired t-tests when applicable. Multiple group comparisons were adjusted using the Benjamini–Hochberg method to control the false discovery rate (FDR).

All statistical analyses in this study were performed using the R programming language (v 4.2.0). Unless otherwise stated, all statistical tests were two-sided, and a P-value < 0.05 was considered statistically significant.

## Results

### Development and validation of NETs-related genetic subtypes

**FIGURE 1:**
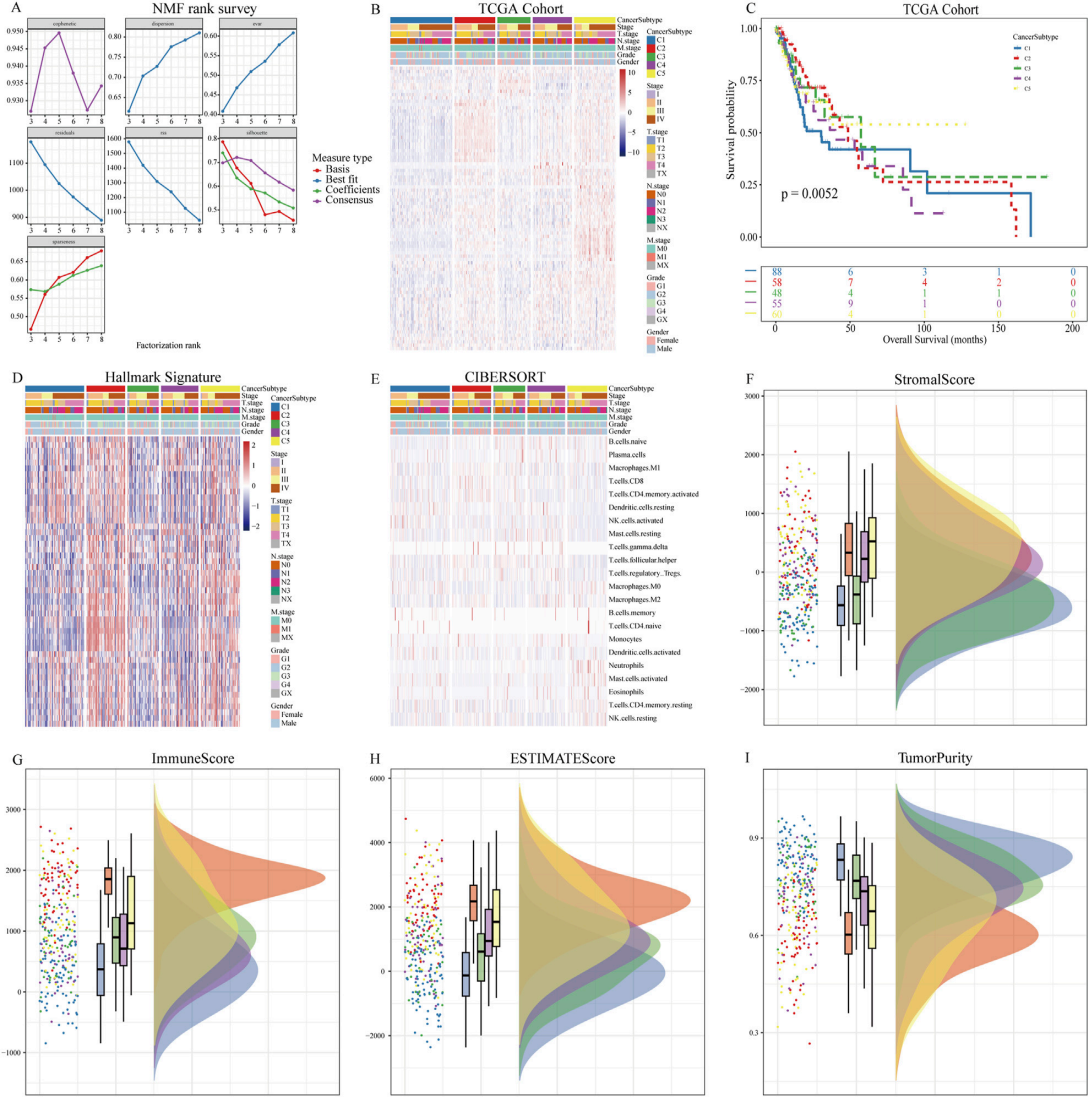
NMF analysis using NETs-related genes. **(A)** Relationship between NMF class coefficients and number of subtypes; **(B)** Heatmap illustrating the distribution of NETs expression in tumor samples across NETs subtypes; **(C)** Kaplan-Meier curves showing OS of patients in different NETs subtypes; **(D)** Differences in hallmark enrichment scores among NETs subtypes; **(E)** Differences in CIBERSORT-related immune cell abundance among NETs subtypes; **(F)** Differences in Stromal Score among NETs subtypes; **(G)** Differences in Immune Score among NETs subtypes; **(H)** Differences in ESTIMATE Score among NETs subtypes; **(I)** Differences in Tumor Purity among NETs subtypes.

Based on the expression levels of 89 NETs-related genes, we conducted NMF analysis on the TCGA cohort. The correlation plot indicated that k = 5 was the optimal number of clusters, resulting in the stratification of OSCC patients into C1-C5 subtypes (Figures 1A,B). Clinical characteristics of patients across the 5 subtypes are summarized in Table 1. There were no significant differences in age, gender, and N stage distribution among the 5 subtypes, while significant differences were observed in UICC stage, T stage, M stage, Grade, and tumor site distribution. Kaplan-Meier survival curve analysis revealed significant differences in overall survival among the 5 subtypes (Figure 1C), with better prognosis observed in C5 subtype patients and shorter survival in C2 and C4 subtype patients. ssGSEA analysis of hallmark pathways showed enrichment of Oxidative_phosphorylation in C1 subtype, Interferon_gamma_response and Interferon_alpha_response in C2 subtype, Kras_signaling in C3 subtype, Adipogenesis in C4 subtype, and Angiogenesis in C5 subtype (Figure 1D). Deconvolution analysis demonstrated enrichment of different immune cells across the 5 subtypes (Figure 1E). Additionally, tumor immune infiltration status was significantly enriched in different subtypes, with higher StromalScore in C5 subtype, higher ImmuneScore and ESTIMATEScore in C2 subtype, and lower TumorPurity in C2 and C5 subtypes. In contrast, C1 subtype exhibited higher TumorPurity (Figures 1F–I).

**TABLE 1:** Clinical characteristics of OSCC patients in the NETs subtype.

Characteristic	C1 (n = 90)	C2 (n = 59)	C3 (n = 48)	C4 (n = 57)	C5 (n = 60)	p value
Gender:						<0.001
Female	24 (26.7%)	29 (49.2%)	21 (43.8%)	8 (14.0%)	17 (28.3%)	
Male	66 (73.3%)	30 (50.8%)	27 (56.2%)	49 (86.0%)	43 (71.7%)	
Age	58.9 (13.1)	65.0 (11.2)	63.3 (12.9)	59.8 (13.7)	62.0 (13.0)	0.039
Tissue:						.
Base of tongue	6 (6.67%)	5 (8.47%)	6 (12.5%)	5 (8.77%)	1 (1.67%)	
Buccal Mucosa	5 (5.56%)	5 (8.47%)	2 (4.17%)	4 (7.02%)	6 (10.0%)	
Floor of mouth	24 (26.7%)	11 (18.6%)	7 (14.6%)	9 (15.8%)	9 (15.0%)	
Hard Palate	1 (1.11%)	1 (1.69%)	3 (6.25%)	0 (0.00%)	2 (3.33%)	
Lip	0 (0.00%)	0 (0.00%)	1 (2.08%)	2 (3.51%)	0 (0.00%)	
Oral Cavity	18 (20.0%)	16 (27.1%)	9 (18.8%)	13 (22.8%)	16 (26.7%)	
Oral Tongue	36 (40.0%)	21 (35.6%)	20 (41.7%)	24 (42.1%)	26 (43.3%)	
Grade:						.
G1	21 (23.9%)	6 (10.2%)	11 (22.9%)	3 (5.26%)	8 (13.3%)	
G2	55 (62.5%)	34 (57.6%)	28 (58.3%)	37 (64.9%)	37 (61.7%)	
G3	11 (12.5%)	18 (30.5%)	8 (16.7%)	12 (21.1%)	15 (25.0%)	
G4	0 (0.00%)	0 (0.00%)	0 (0.00%)	2 (3.51%)	0 (0.00%)	
GX	1 (1.14%)	1 (1.69%)	1 (2.08%)	3 (5.26%)	0 (0.00%)	
HPV.status:						0.011
Negative	11 (91.7%)	8 (100%)	6 (60.0%)	10 (71.4%)	17 (100%)	
Positive	1 (8.33%)	0 (0.00%)	4 (40.0%)	4 (28.6%)	0 (0.00%)	
Smoking:						0.698
No	44 (48.9%)	31 (52.5%)	23 (47.9%)	23 (40.4%)	26 (43.3%)	
Yes	46 (51.1%)	28 (47.5%)	25 (52.1%)	34 (59.6%)	34 (56.7%)	
Alcohol:						0.071
No	33 (37.9%)	25 (42.4%)	17 (36.2%)	12 (22.6%)	14 (23.3%)	
Yes	54 (62.1%)	34 (57.6%)	30 (63.8%)	41 (77.4%)	46 (76.7%)	
Stage:						.
I	1 (1.11%)	3 (5.08%)	3 (6.25%)	3 (5.26%)	2 (3.33%)	
II	24 (26.7%)	22 (37.3%)	11 (22.9%)	15 (26.3%)	9 (15.0%)	
III	17 (18.9%)	9 (15.3%)	16 (33.3%)	18 (31.6%)	11 (18.3%)	
IV	48 (53.3%)	25 (42.4%)	18 (37.5%)	21 (36.8%)	38 (63.3%)	
T.stage:						.
T1	4 (4.44%)	4 (6.78%)	4 (8.33%)	6 (10.5%)	3 (5.00%)	
T2	31 (34.4%)	24 (40.7%)	17 (35.4%)	19 (33.3%)	14 (23.3%)	
T3	25 (27.8%)	13 (22.0%)	11 (22.9%)	15 (26.3%)	17 (28.3%)	
T4	28 (31.1%)	17 (28.8%)	14 (29.2%)	15 (26.3%)	26 (43.3%)	
TX	2 (2.22%)	1 (1.69%)	2 (4.17%)	2 (3.51%)	0 (0.00%)	
N.stage:						.
N0	40 (44.4%)	34 (57.6%)	24 (50.0%)	31 (54.4%)	29 (48.3%)	
N1	16 (17.8%)	11 (18.6%)	12 (25.0%)	9 (15.8%)	9 (15.0%)	
N2	29 (32.2%)	12 (20.3%)	8 (16.7%)	14 (24.6%)	21 (35.0%)	
N3	1 (1.11%)	1 (1.69%)	0 (0.00%)	0 (0.00%)	1 (1.67%)	
NX	4 (4.44%)	1 (1.69%)	4 (8.33%)	3 (5.26%)	0 (0.00%)	
M.stage:						0.714
M0	83 (92.2%)	58 (98.3%)	46 (95.8%)	53 (93.0%)	58 (96.7%)	
M1	2 (2.22%)	0 (0.00%)	0 (0.00%)	0 (0.00%)	0 (0.00%)	
MX	5 (5.56%)	1 (1.69%)	2 (4.17%)	4 (7.02%)	2 (3.33%)	

### WGCNA network identifies subtype-associated genes

**FIGURE 2:**
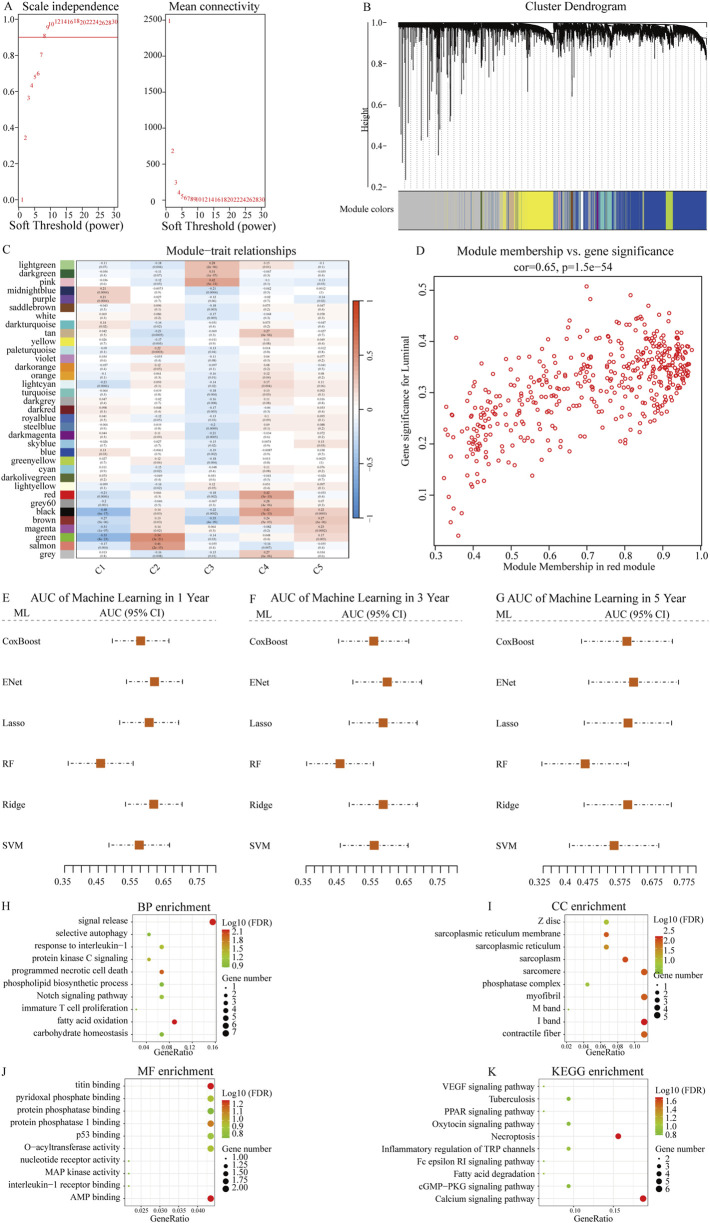
Identification of NETs-related prognostic genes using WGCNA and machine learning. **(A)** Scale-free topology fit index (left) and mean connectivity (right) for various soft-thresholding powers in WGCNA. Power = 8 was selected to ensure approximate scale-free topology (R^2^ = 0.9); **(B)** Dendrogram of gene clustering based on topological overlap matrix. Different modules are represented by distinct colors; **(C)** Heatmap showing the correlation between each module eigengene and NETs molecular subtypes; **(D)** Scatter plot showing the correlation between module membership and gene significance for the red module (cor = 0.65, p = 1.5e−54), indicating that key genes within the module are closely associated with subtype traits; **(E–G)** Comparison of predictive performance of six machine learning algorithms in OSCC prognosis prediction. Time-dependent AUC values at 1 year, 3 years, and 5 years were calculated using cross-validation. Higher AUC values indicate better predictive accuracy. The Enet model achieved the best overall performance and was selected to construct the final NETs-related prognostic signature; **(H–K)** Bubble plots showing enrichment analysis results of NETs signature-related genes in BP (biological process), CC (cellular component), MF (molecular function), and KEGG (Kyoto Encyclopedia of Genes and Genomes) pathways.

After constructing the co-expression network using WGCNA with a soft-thresholding power of β = 8, we identified multiple gene modules based on topological overlap and hierarchical clustering (R^2^ = 0.9, Figures 2A,B). To determine the biologically relevant module, we evaluated the correlation between module eigengenes (MEs) and the defined NETs subtypes (Figure 2C). The red module showed the strongest and most statistically significant correlation (cor = 0.65, p = 1.5e–54, Figure 2D), indicating a meaningful association with NETs-driven phenotypes. Genes within this module were further filtered using univariate Cox regression to identify those significantly associated with overall survival (p < 0.01, Table 2). This process yielded 186 candidate genes for subsequent machine learning-based model construction. Next, we applied six machine learning algorithms to filter the 186 genes and select the optimal NETs signature. We calculated the AUC values of each model for 1-, 3-, and 5-year survival to evaluate their prognostic performance in OSCC patients. The results showed that the Enet model identified 52 genes with the highest AUC values (Figures 2E–G; Table 2; Supplementary Table 1).

**TABLE 2:** The univariate Cox analysis of genes in Enet model.

Gene	HR	CI5	CI95	p value	CI 95%
CASQ2	0.25	0.11	0.57	0.001	0.11–0.57
MAPK13	0.35	0.15	0.80	0.012	0.15–0.8
ETFDH	0.37	0.20	0.68	0.002	0.2–0.68
GOT1	0.40	0.27	0.60	0.000	0.27–0.6
SYN1	0.43	0.25	0.73	0.002	0.25–0.73
ACADM	0.43	0.23	0.78	0.006	0.23–0.78
PKIA	0.44	0.20	0.95	0.036	0.2–0.95
SLC25A4	0.45	0.29	0.71	0.001	0.29–0.71
ZNF385B	0.46	0.28	0.76	0.003	0.28–0.76
SERPINB5	0.46	0.25	0.85	0.012	0.25–0.85
DCAF6	0.47	0.23	0.95	0.036	0.23–0.95
PFKM	0.49	0.32	0.76	0.002	0.32–0.76
DGLUCY	0.49	0.30	0.78	0.003	0.3–0.78
GPD1L	0.51	0.35	0.74	0.000	0.35–0.74
RNF149	0.51	0.35	0.76	0.001	0.35–0.76
PPP1R3F	0.53	0.36	0.79	0.001	0.36–0.79
NNAT	0.53	0.29	0.97	0.039	0.29–0.97
SCT	0.53	0.28	0.99	0.046	0.28–0.99
CLTCL1	0.54	0.37	0.80	0.002	0.37–0.8
YBX3	0.54	0.36	0.82	0.004	0.36–0.82
LINC00937	0.55	0.36	0.83	0.005	0.36–0.83
CRAT	0.55	0.35	0.85	0.008	0.35–0.85
PLN	0.55	0.35	0.88	0.012	0.35–0.88
IDH2	0.55	0.33	0.94	0.027	0.33–0.94
CAMK2A	0.56	0.35	0.90	0.016	0.35–0.9
ANKRD10	0.56	0.32	0.99	0.048	0.32–0.99
MYLK4	0.57	0.35	0.93	0.024	0.35–0.93
TRIM63	0.58	0.38	0.87	0.009	0.38–0.87
P2RX6	0.58	0.38	0.91	0.016	0.38–0.91
ACYP2	0.58	0.37	0.91	0.017	0.37–0.91
PYGL	0.59	0.39	0.90	0.013	0.39–0.9
TMEM38B	0.60	0.40	0.88	0.009	0.4–0.88
ACTC1	0.60	0.37	0.95	0.031	0.37–0.95
SMTNL2	0.61	0.42	0.88	0.009	0.42–0.88
IP6K3	0.63	0.43	0.93	0.021	0.43–0.93
PPP1R3C	0.63	0.41	0.96	0.033	0.41–0.96
MAP3K7CL	0.64	0.44	0.93	0.019	0.44–0.93
CIDEC	0.64	0.44	0.94	0.023	0.44–0.94
KLHL30	0.64	0.43	0.95	0.026	0.43–0.95
ADH1B	0.66	0.45	0.95	0.027	0.45–0.95
ADIPOQ	0.67	0.46	0.98	0.041	0.46–0.98
ANKRD1	0.69	0.47	1.00	0.049	0.47–1
SVIL2P	1.48	1.02	2.15	0.041	1.02–2.15
VIT	1.55	1.04	2.31	0.030	1.04–2.31
GRIP2	1.60	1.10	2.32	0.013	1.1–2.32
NATD1	1.79	1.14	2.80	0.011	1.14–2.8
AC104564.1	1.95	1.23	3.09	0.004	1.23–3.09
HSPB8	2.17	1.05	4.47	0.036	1.05–4.47
SCN3B	2.36	1.26	4.41	0.007	1.26–4.41
ASB1	2.71	1.10	6.65	0.030	1.1–6.65
PLA2G4C	2.74	1.20	6.24	0.017	1.2–6.24
FAM238C	3.45	1.80	6.62	0.000	1.8–6.62

Subsequently, we conducted GO enrichment analysis and KEGG pathway analysis on the genes identified by the Enet model. GO enrichment analysis revealed that these feature genes were enriched in fatty acid oxidation (BP, Figure 2H; Supplementary Table 2), contractile fiber (CC, Figure 2I; Supplementary Table 2), and AMP binding (MF, Figure 2J; Supplementary Table 2). KEGG pathway analysis indicated enrichment in Necroptosis for these feature genes (Figure 2K; Supplementary Table 3).

### Determination and validation of a prognostic NETs signature

**FIGURE 3:**
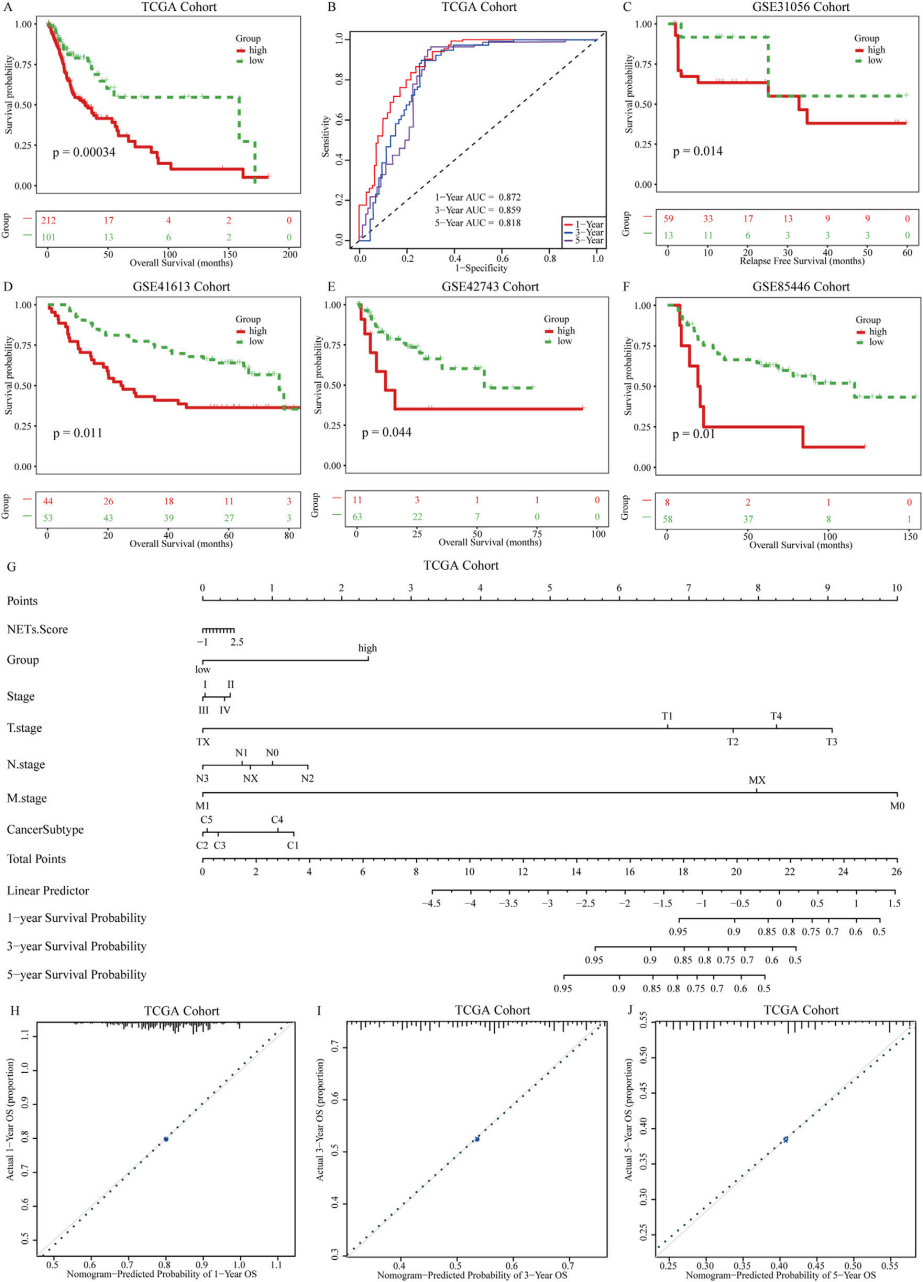
Establishment of prognostic NETs signature for OSCC. **(A)** Kaplan-Meier curves of OS for patients grouped by NETs signature; **(B)** AUC values for the NETs signature predicting 1 years, 3 years, and 5 years survival in OSCC patients; **(C–F)** Kaplan-Meier curves of OS for patients grouped by NETs signature in four validation cohorts: GSE31056, GSE31056, GSE42743, and GSE85446; **(G)** Nomogram model constructed with the NETs signature; **(H–J)** Calibration plots of the nomogram model showing the consistency between predicted and actual survival rates at 1 year, 3 years, and 5 years.

Based on ssGSEA, we calculated the NESs of the feature genes identified by the Enet model in OSCC patients, defining it as the NETs signature. Using the optimal cutoff value based on the NETs signature, we divided patients into high and low NETs signature groups. We first evaluated the differences in clinical characteristics between the high and low NETs signature groups (Table 3). Notably, the low NETs signature group had a higher proportion of HPV-positive patients, while the high NETs signature group had a higher proportion of smokers. Additionally, stage IV patients were predominantly enriched in the low NETs signature group. Further analysis of their impact on OSCC prognosis revealed that patients in the low NETs signature group had significantly longer survival times compared to those in the high NETs signature group (Figure 3A). Moreover, ROC curve analysis demonstrated that the NETs signature had high predictive efficacy for 1-, 3-, and 5-year prognosis in OSCC patients (Figure 3B). To ensure the robustness of our findings, we performed further validation using four validation cohorts. The results showed consistent favorable outcomes for the NETs signature across all four validation cohorts (Figures 3C–F).

**TABLE 3:** Clinical characteristics of OSCC patients in the NETs signature.

Characteristic	High (n = 213)	low (n = 101)	p value
Gender:			0.0727
Female	69 (32.4%)	30 (29.7%)	
Male	144 (67.6%)	71 (70.3%)	
Age	60.1 (13.6)	64.3 (11.0)	0.0004
Tissue:			0.0504
Base of tongue	12 (5.63%)	11 (10.9%)	
Buccal Mucosa	15 (7.04%)	7 (6.93%)	
Floor of mouth	33 (15.5%)	27 (26.7%)	
Hard Palate	1 (0.47%)	6 (5.94%)	
Lip	1 (0.47%)	2 (1.98%)	
Oral Cavity	44 (20.7%)	28 (27.7%)	
Oral Tongue	107 (50.2%)	20 (19.8%)	
Grade:			0.0293
G1	32 (15.0%)	17 (17.2%)	
G2	137 (64.3%)	54 (54.5%)	
G3	38 (17.8%)	26 (26.3%)	
G4	1 (0.47%)	1 (1.01%)	
GX	5 (2.35%)	1 (1.01%)	
HPV.status:			0.015
Negative	40 (93.0%)	12 (66.7%)	
Positive	3 (6.98%)	6 (33.3%)	
Smoking:			0.0307
No	95 (44.6%)	52 (51.5%)	
Yes	118 (55.4%)	49 (48.5%)	
Alcohol:			0.0764
No	67 (32.2%)	34 (34.7%)	
Yes	141 (67.8%)	64 (65.3%)	
Stage:			0.0458
I	10 (4.69%)	2 (1.98%)	
II	57 (26.8%)	24 (23.8%)	
III	50 (23.5%)	21 (20.8%)	
IV	96 (45.1%)	54 (53.5%)	
T.stage:			0.0527
T1	15 (7.04%)	6 (5.94%)	
T2	76 (35.7%)	29 (28.7%)	
T3	56 (26.3%)	25 (24.8%)	
T4	62 (29.1%)	38 (37.6%)	
TX	4 (1.88%)	3 (2.97%)	
N.stage:			0.0538
N0	105 (49.3%)	53 (52.5%)	
N1	41 (19.2%)	16 (15.8%)	
N2	59 (27.7%)	25 (24.8%)	
N3	1 (0.47%)	2 (1.98%)	
NX	7 (3.29%)	5 (4.95%)	
M.stage:			0.0203
M0	205 (96.2%)	93 (92.1%)	
M1	1 (0.47%)	1 (0.99%)	
MX	7 (3.29%)	7 (6.93%)	
CancerSubtype:			0.0463
C1	60 (28.2%)	30 (29.7%)	
C2	40 (18.8%)	19 (18.8%)	
C3	28 (13.1%)	20 (19.8%)	
C4	40 (18.8%)	17 (16.8%)	
C5	45 (21.1%)	15 (14.9%)	

To provide clinicians with a quantifiable method for patient prognosis, we further explored the potential associations between the NETs signature and clinical pathological features in the TCGA cohort. We constructed a nomogram model (Figure 3G) incorporating the NETs signature and clinical pathological features. Higher scores of the NETs signature in OSCC patients were associated with poorer prognosis. Based on the calibration curves of the nomogram model, we used the NETs signature to predict the survival probabilities of patients at 1, 3, and 5 years after diagnosis of OSCC. The calibration curves for 1-, 3-, and 5-year survival probabilities accurately predicted the survival rates of patients at these time points (Figures 3H–J). These results demonstrate that the nomogram model based on the NETs signature has strong discriminatory and calibration abilities.

### Features of NETs signatures in tumor microenvironment

**FIGURE 4:**
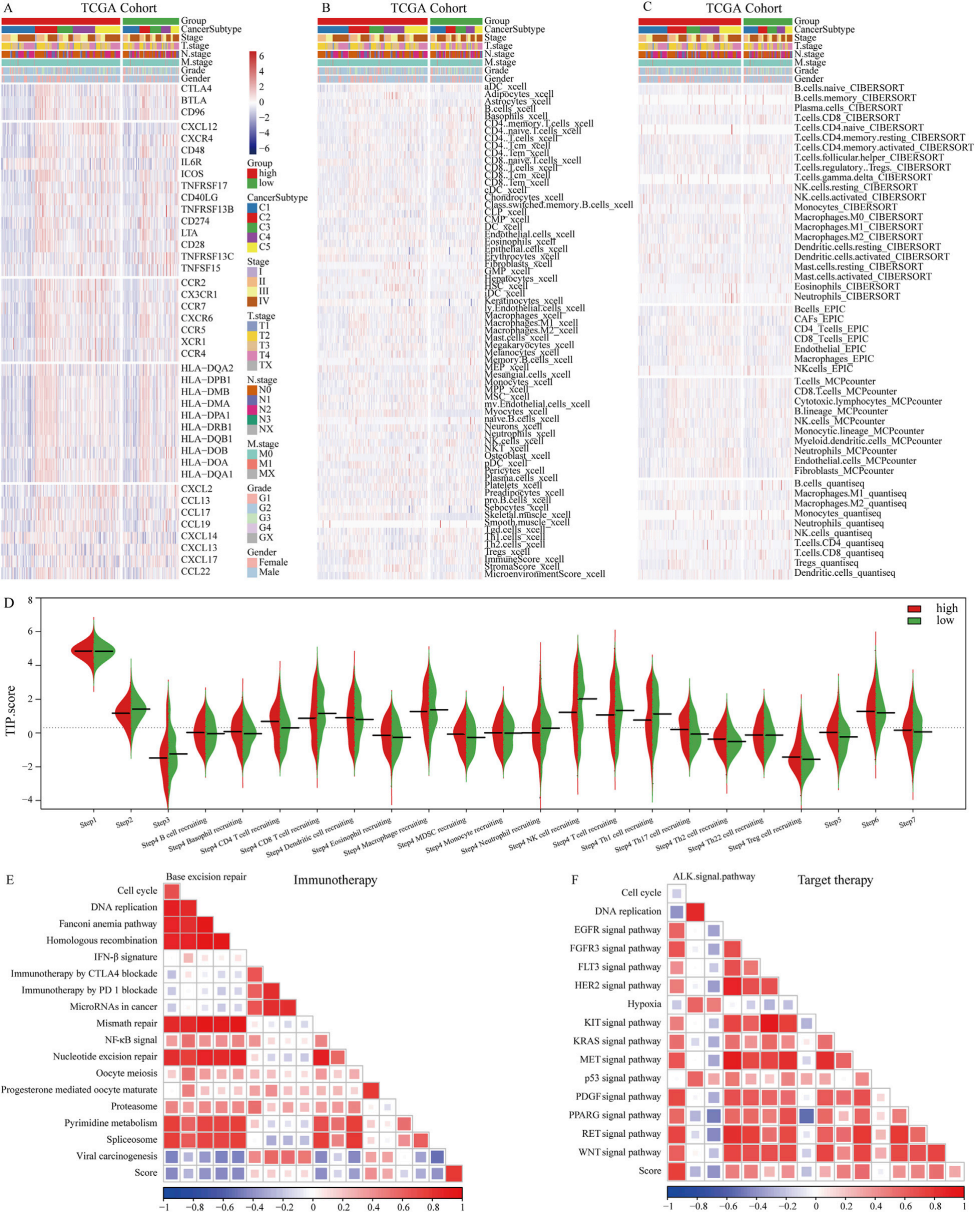
Immune characteristics of the NETs signature. **(A)** Heatmap showing the correlation between the NETs signature and 5 classes of immune regulatory molecules; **(B)** Heatmap illustrating differences between NETs signature and immune infiltrating cells using the Xcell algorithm; **(C)** Heatmap demonstrating differences between NETs signature and immune infiltrating cells using QUANTISEQ, CIBERSORT, MCPCOUNTER, and EPIC algorithms; **(D)** Bean plot showing differences between NETs signature and TIP score; **(E)** Heatmap showing the correlation between the NETs signature in OSCC and immune-related pathways; **(F)** Heatmap showing the correlation between the NETs signature in OSCC and targeted therapy-related pathways.

In patients with OSCC, the NETs signature shows significant correlations with 5 categories of immune regulatory factors (Figure 4A). Antigen presentation, immune activation, and receptor molecules are particularly highly expressed in the high NETs signature group, whereas chemokines and immune suppression factors are highly expressed in the low NETs signature group. Various deconvolution algorithms were used to assess the immune cell infiltration density between the two patient groups. In the high NETs signature group, OSCC patients enrich immune-promoting cells such as NK cells, CD8^+^ T cells, and CD4^+^ T cells, while in the low NETs signature group, immune-suppressive cells such as myeloid-derived suppressor cells (MDSCs), neutrophils, mast cells, and fibroblasts are enriched (Figures 4B,C). We used an online tool to calculate the TIP score of OSCC patients to explore the biological mechanisms related to the NETs signature. Cancer immune cycle is more activated in the low NETs signature group, including cancer antigen presentation (Step 2), immune cell activation (Step 3), and recruitment of tumor immune infiltrating cells (Step 4). In contrast, immune cell infiltration (Step 5), T cell recognition of tumor cells (Step 6), and killing of tumor cells (Step 7) are enriched in the high NETs signature group (Figure 4D). Additionally, the NETs signature is significantly negatively correlated with PD-1 immune therapy, nucleotide excision repair, and mismatch repair (Figure 4E). We also observed significant positive correlations between the NETs signature and the ALK signaling pathway, FGFR3 signaling pathway, while negative correlations were found with the EGFR signaling pathway and KIT signaling pathway (Figure 4F).

### NETs related immune features

**FIGURE 5:**
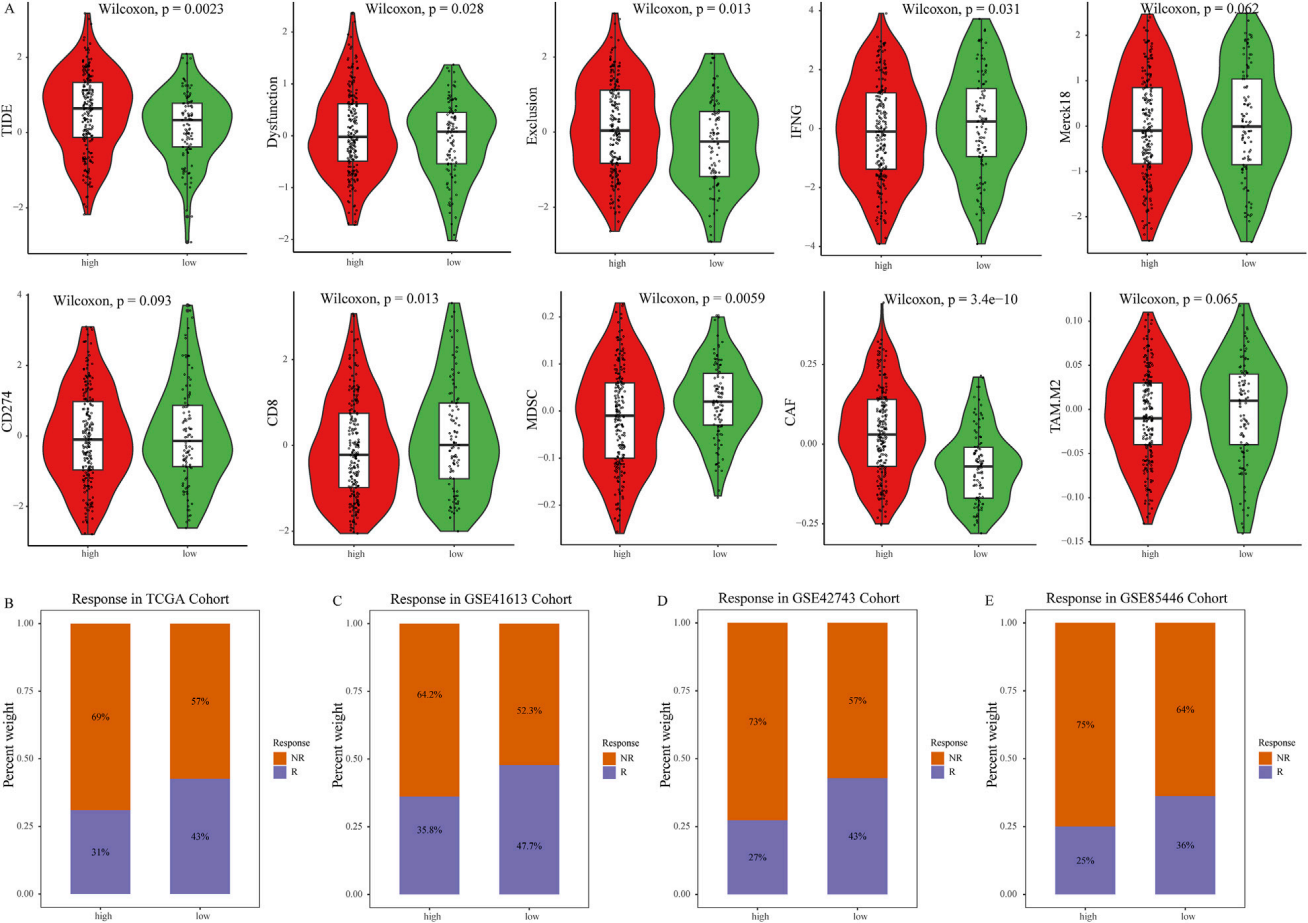
Predictive value of the NETs signature for immunotherapy. **(A)** Violin plot showing TIDE score, Exclusion score, Dysfunction score, IFNG level, Merck18 score, CD274 score, CD8 score, MDSC score, CAF score, and tumor-associated M2 macrophage score between the two NETs signature groups; **(B)** Bar graph showing the number of responders to immunotherapy in each NETs signature group; **(C–E)** Bar graphs showing the number of responders to immunotherapy in four validation cohorts, namely, GSE31056, GSE31056, GSE42743, and GSE85446 cohorts.

We further explored the relationship between the NETs signature and various immune therapy predictive factors. TIDE score, Dysfunction score, Exclusion score, CD274, and CAF are higher in the high NETs signature group (Figure 5A). Conversely, IFNG score, Merck18, myeloid-derived suppressor cells (MDSCs), CD8, and tumor-associated M2 macrophages are higher in the low NETs signature group (Figure 5A). We also analyzed the association between the NETs signature and immune therapy response rates. The results revealed a higher proportion of immune therapy response in the low NETs signature group (Figure 5B). The NETs signature was further analyzed in multiple validation cohorts. It is evident that the low NETs signature group exhibits higher response rates to immune therapy, while the high NETs signature group is relatively resistant (Figures 5C–F). Based on the TIDE algorithm, OSCC patients in the low NETs signature group have a better response to immune therapy.

### LINC00937 is highly expressed in OSCC

**FIGURE 6:**
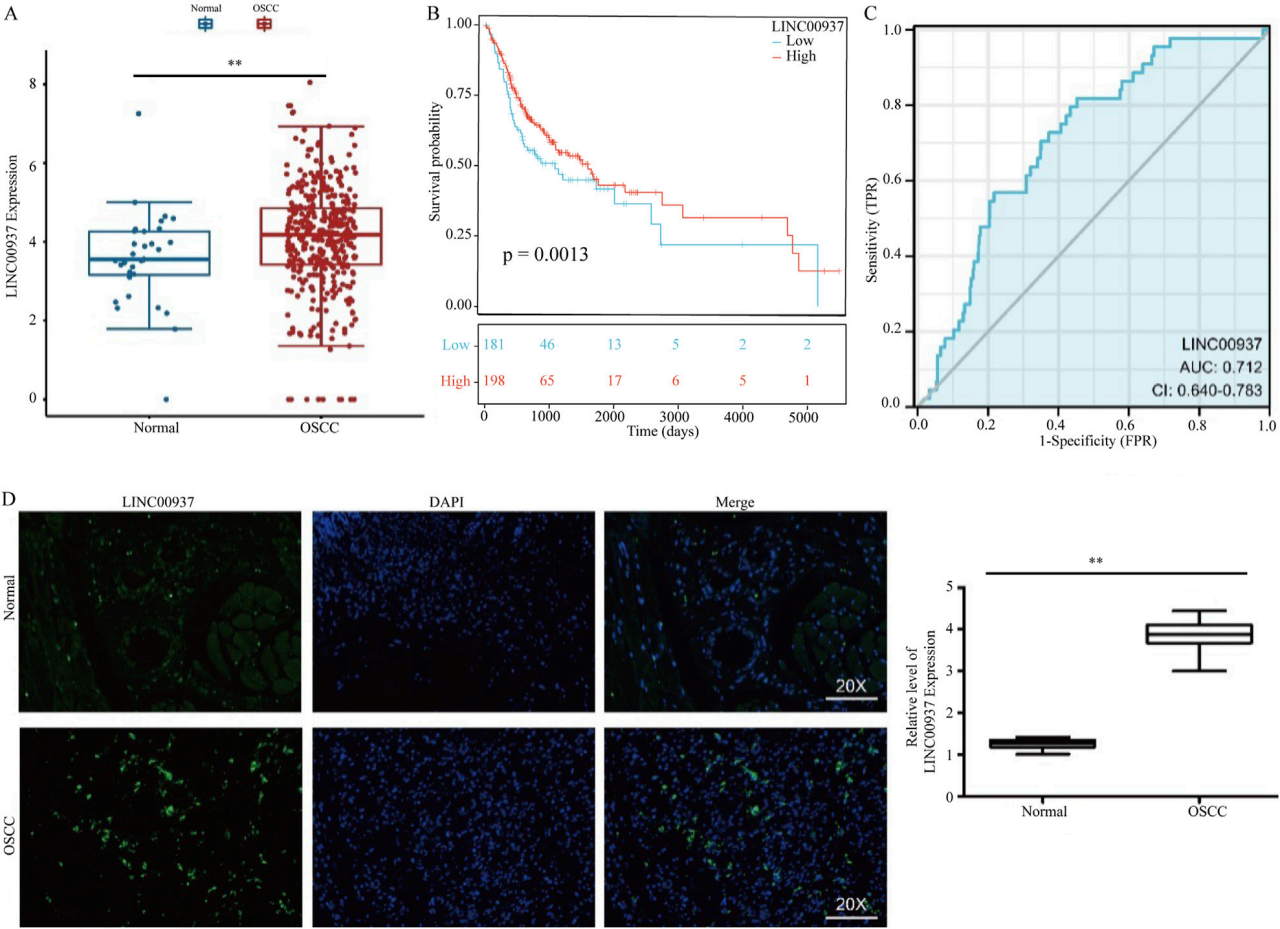
LINC00937 has a prognostic value in OSCC. **(A)** the expression of LINC00937 between OSCC tissues and adjacent tissues from TCGA cohort; **(B)** Kaplan-Meier analysis of LINC00937 in OSCC by TCGA cohort; **(C)** AUC area of LINC00937 in TCGA cohort; **(D)** FISH staining was used to show the expression and location in OSCC patients. ** represents p < 0.01.

Due to its higher expression levels and survival relevance in OSCC patients, we selected LINC00937 for further analysis (Figures 6A–C). Compared to normal tissue samples, LINC00937 is upregulated in OSCC tumor tissue samples (p = 0.007) (Figure 6A). Using the optimal cutoff value of LINC00937 expression levels, OSCC patients were grouped into high and low expression groups, where patients with high expression levels had longer survival times compared to those with low expression levels (Figure 6B). Subsequent ROC analysis indicated that LINC00937 has a strong discriminatory ability between tumor and normal tissues (AUC = 0.712, Figure 6C). FISH was used to detect the levels and localization of LINC00937 in OSCC tissues. Our experimental results showed that in 10 pairs of OSCC tumor tissue samples and normal tissue samples, LINC00937 is more highly expressed in OSCC tumor tissue samples and is localized in the cell nucleus (Figure 6D).

**FIGURE 7:**
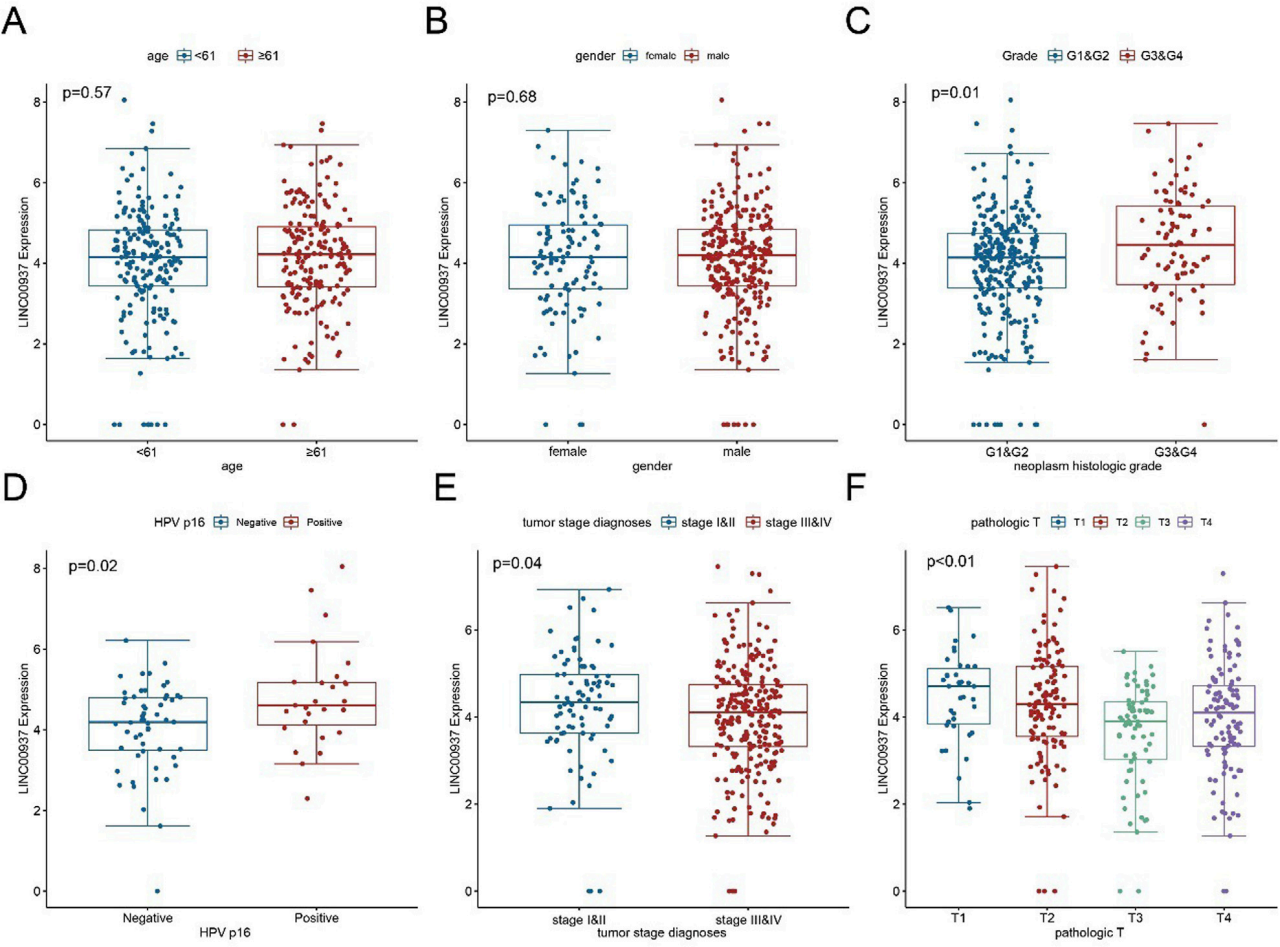
The association between LINC00937 and clinicopathological characteristics of OSCC. **(A)** Comparison of LINC00937 expression levels in OSCC patients grouped by age based on TCGA cohort; **(B)** Comparison of LINC00937 expression levels in OSCC patients grouped by gender based on TCGA cohort; **(C)** Comparison of LINC00937 expression levels in OSCC patients grouped by tumor grade based on TCGA database; **(D)** Comparison of LINC00937 expression levels in OSCC patients grouped by HPV status based on TCGA cohort; **(E)** Comparison of LINC00937 expression levels in OSCC patients grouped by tumor stage based on TCGA cohort; **(F)** Comparison of LINC00937 expression levels in OSCC patients grouped by T stage based on TCGA cohort. * represents p < 0.05, ** represents p < 0.01, *** represents p < 0.001.

We then evaluated the relationship between clinical pathological features and the expression levels of LINC00937. The expression level of LINC00937 was significantly associated with tumor grade (p = 0.01), HPV status (p = 0.02), pathological stage (p = 0.04), while it showed no association with age (p = 0.57) and gender (p = 0.68) (Figures 7A–F). Meanwhile, LINC00937 exhibits positive correlations with several NETs-related genes, such as DCAF6, PKIA, and KLHL30, and negative correlations with other genes. Most correlations were in the mild-to-moderate positive range, suggesting that high LINC00937 expression may be associated with an overall transcriptional profile enriched for NETs activity (Supplementary Figure S1).

### LINC00937 promotes proliferation and migration of OSCC cells

**FIGURE 8:**
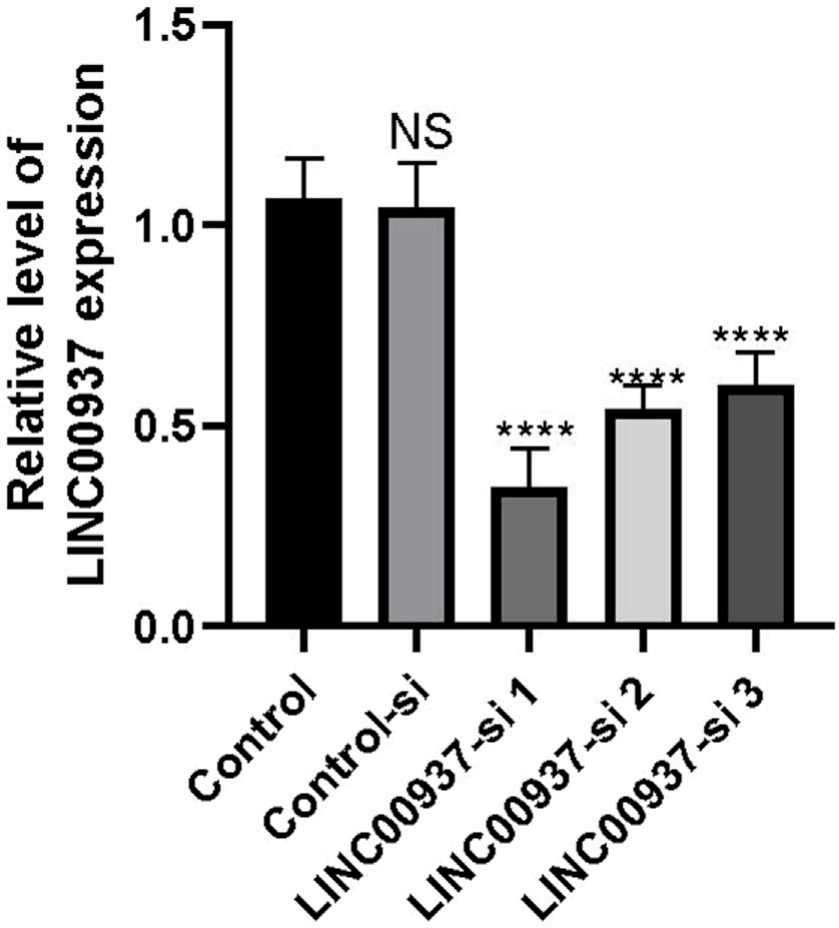
The difference in LINC00937 expression levels before and after knockdown (the number of biological replicates is three independent experiments), *p < 0.05, ****p < 0.001.

**FIGURE 9:**
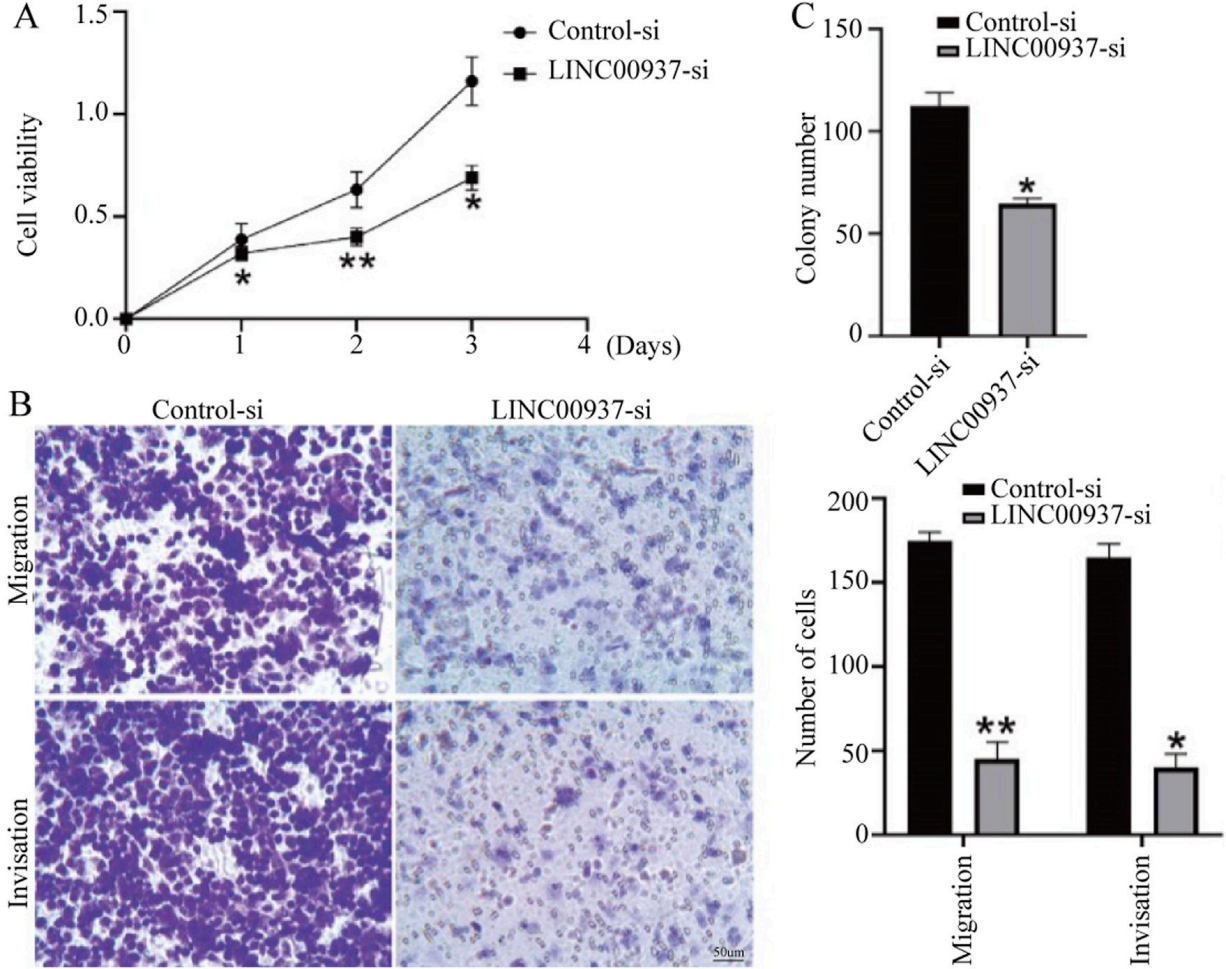
The biological functions of LINC00937 in the proliferation and metastasis of OSCC cells. **(A)** CCK-8 assay shows that knockdown of LINC00937 results in reduced growth of OSCC cells. **(B)** Knockdown of LINC00937 inhibits the migration and invasion of CAL-27 cells. **(C)** Colony formation assay to detect the number of cell colonies. *, p < 0.05, **, p < 0.01.

We found that the expression level of LINC00937 is significantly elevated in OSCC tumor tissues, and LINC00937 can impact the development of OSCC. To further evaluate the significance of LINC00937 in OSCC, we used siRNA tools to construct a stable CAL-27 cell line with LINC00937 knocked down (Figure 8). Firstly, in CAL-27 cells, silencing LINC00937 inhibited cell growth compared to the control group (Figure 9A). Secondly, trans-well experiments showed that knocking down LINC00937 expression suppressed the migration and invasion of OSCC cells (Figure 9B). Thirdly, inhibiting LINC00937 suppressed the proliferative ability of OSCC cells, as shown by the colony formation assay (Figure 9C). Our study results indicate that inhibiting the expression of LINC00937 *in vitro* reduces OSCC cell proliferation, induces apoptosis, and suppresses metastasis, thereby affecting the occurrence and development of OSCC.

## Discussion

As key mediators involved in extracellular matrix formation, angiogenesis, and immune response, NETs play a critical role in tumor progression and metastasis. NETs-related genes have been identified as promising therapeutic targets in various cancers. Therefore, establishing a robust prognostic signature and exploring genes that mediate NETs formation may offer new therapeutic strategies for OSCC.

Based on previous studies of NETs-related genes, this study identified five subtypes in patients with OSCC. Significant differences were observed in tumor staging among the five subtypes, with subtype C5 associated with better prognosis and subtype C2/C4 with poorer prognosis. To ensure robustness and generalizability of the prognostic NETs signature, we adopted a machine learning framework incorporating six widely used algorithms. These models were selected based on their complementary strengths in handling high-dimensional, multicollinear, and survival-related data. Feature selection was performed through the embedded regularization techniques or variable importance scores within each model—such as L1 or L2 penalties in Lasso and Enet, or feature importance ranking in Random Forest. This approach allowed us to minimize overfitting while retaining the most informative genes. Among all models tested, the Enet model achieved the best predictive performance across multiple time points (1-, 3-, and 5-year AUCs) and was therefore selected for final signature construction. This multi-model strategy enhances the stability and reliability of the prognostic biomarker.

High NETs signature group patients exhibit a significant presence of anti-tumor immune cells in the TME, such as NK cells (Myers et al., 2021; Tang et al., 2023), CD8^+^ T cells (Virassamy et al., 2023), and CD4^+^ T cells (Li et al., 2024). Conversely, in the low NETs signature group, OSCC patients enrich immune-suppressive cells like MDSCs (Hegde et al., 2021; Cassetta et al., 2020), mast cells (Cheng et al., 2021), fibroblasts (Caligiuri and Tuveson, 2023; Biffi and Tuveson, 2021), and granulocytes. Additionally, various immune modulators including antigen presentation, immune inhibition, immune activation, chemokines, and receptors are upregulated in the high NETs signature group, suppressing tumor cell recurrence and metastasis. Furthermore, cancer immune cycles are more activated in the low NETs signature group. All these factors seem to imply that patients in the high NETs signature group should have a better prognosis. However, in our study, patients in the low NETs signature group had better outcomes. We need to further explore the mechanisms underlying this contradiction in future research.

From the perspective of immune therapy, the NETs signature can predict response rates in patients with OSCC receiving anti-PD-1 or anti-PD-L1 therapy. It is noteworthy that patients in the high NETs signature group benefit less from immune therapy. Some immune suppression markers are upregulated in the high NETs signature group, suggesting a potential link to lower response rates. Therefore, improving the expression levels of these immune suppression markers in the tumor microenvironment of the high NETs signature group should be a major therapeutic focus.

In our study, we observed that OSCC patients with a low NETs signature had significantly better overall survival and showed enhanced responsiveness to immunotherapy. This finding appears to contradict certain prior reports suggesting that NETs can exert anti-tumorigenic effects by enhancing immune surveillance or trapping tumor cells. To reconcile this discrepancy, we propose that the role of NETs in cancer may be highly context-dependent. In the case of OSCC, it is plausible that the immunosuppressive effects of NETs, such as recruitment of MDSCs, increased expression of PD-L1, and inhibition of cytotoxic T cell activity, outweigh any potential anti-tumor mechanisms. Moreover, it is important to note that the NETs signature developed in this study was based on a gene expression-based machine learning model and does not exclusively represent classical NETs structural proteins. Instead, it may reflect broader transcriptomic changes associated with a NET-rich, immune-suppressive, or stromal-activated tumor microenvironment. Therefore, the high NETs signature may indirectly indicate a more immunosuppressive tumor state, explaining its association with worse prognosis and reduced immunotherapy benefit. These insights highlight the complexity of NETs biology in OSCC and underscore the need for mechanistic studies at both the cellular and pathway levels.

Although previous studies, such as Zhang et al. (2022), have proposed NETs-related scoring systems in a pan-cancer context, our study provides several key innovations in the OSCC-specific setting. First, we applied unsupervised clustering based on NETs-related genes to define molecular subtypes with distinct immune phenotypes and prognoses in OSCC, which has not been previously reported. Second, we constructed a robust prognostic signature through systematic machine learning involving six different algorithms, allowing model comparison and optimal selection based on cross-validated AUC performance. Importantly, our model was trained and validated specifically in OSCC, rather than generalized across cancers, thus increasing its disease relevance. Furthermore, our study bridges bioinformatic prediction and biological validation by identifying LINC00937 as a NETs-signature-derived oncogene and confirming its pro-tumorigenic role *in vitro*. Together, these contributions enhance both the clinical utility and mechanistic understanding of NETs in OSCC, offering a complementary and novel perspective beyond previous pan-cancer frameworks.

Previous studies have revealed that LINC00937 acts as an oncogenic factor in certain cancers, such as cutaneous melanoma (Xu S. et al., 2018). In our study, we found that LINC00937 is highly expressed in tumor tissues of OSCC patients. When we correlated LINC00937 expression with TNM staging in OSCC patients, we also found that LINC00937 is lowly expressed in late-stage patients, further demonstrating its impact on the prognosis of OSCC patients. *In vitro* experiments showed that knockdown of LINC00937 in CAL-27 cell lines inhibits cell proliferation, induces apoptosis, and suppresses migration and invasion. Based on these results, we speculate that LINC00937 is involved in the regulation of pathological progression in OSCC.

Interestingly, we observed a seeming contradiction between clinical and experimental data regarding LINC00937. Clinically, higher expression of LINC00937 was associated with improved overall survival and lower tumor stage in OSCC patients, suggesting a potential tumor-suppressive role. However, *in vitro* experiments showed that knockdown of LINC00937 suppressed proliferation, migration, and invasion of OSCC cells, indicating an oncogenic function. This discrepancy may be attributed to several factors. One possible explanation lies in the complexity of the tumor microenvironment (TME). The LINC00937 expression detected in bulk tumor transcriptomes may originate not only from malignant epithelial cells but also from stromal or immune components. If LINC00937 is predominantly expressed in non-tumor cells such as immune or fibroblast populations, its higher expression could reflect a less aggressive or more immune-responsive tumor phenotype, which aligns with better clinical outcomes. In contrast, our *in vitro* findings specifically reflect the function of LINC00937 in tumor epithelial cells, isolated from the broader TME, where it appears to facilitate oncogenic behavior. Another possibility is that LINC00937 may exert context-dependent, dual functions depending on the cellular environment, epigenetic state, or molecular subtype. Similar contradictory roles have been documented for other long non-coding RNAs in cancer. Therefore, further mechanistic studies—such as single-cell RNA sequencing or spatial transcriptomics—are warranted to delineate the cellular sources and context-specific functions of LINC00937 in OSCC.

This study has several limitations. Firstly, it is based on publicly available bulk data, which cannot fully reflect the cell-cell interaction effects of neutrophils and other immune cells. Due to the short lifespan of neutrophils, single-cell sequencing faces challenges in sample acquisition, and the sequencing depth is relatively low. Additionally, although this study revealed the association between LINC00937 and NETs formation, its underlying mechanisms still need further validation at the pathway level.

## Conclusion

As described above, our findings suggest that NETs play a critical role in the tumor progression of OSCC. We conducted NMF analysis on OSCC patients and identified five subtypes associated with NETs. Using various machine learning algorithms, this study established and validated a robust NETs signature for OSCC patients. Subsequently, LINC00937 was identified as a key gene and further investigated through *in vitro* experiments. Ultimately, we demonstrated that LINC00937 plays a detrimental role in OSCC tumor growth and is involved in the regulation of OSCC pathogenesis.

## Data Availability

Data for this study were sourced from publicly accessible databases, including TCGA (www.portal.gdc.cancer.gov/), GEO (www.ncbi.nlm.nih.gov/geo/). The R analysis code is available upon request.
