# Supplementary figures and images for: Constructing a neutrophil extracellular trap model based on machine learning to predict clinical outcomes and immune therapy responses in oral squamous cell carcinoma
Source: Front Genet. 2025 Sep 8;16:1616868. doi: 10.3389/fgene.2025.1616868 (PMC12450687; doi:10.3389/fgene.2025.1616868)

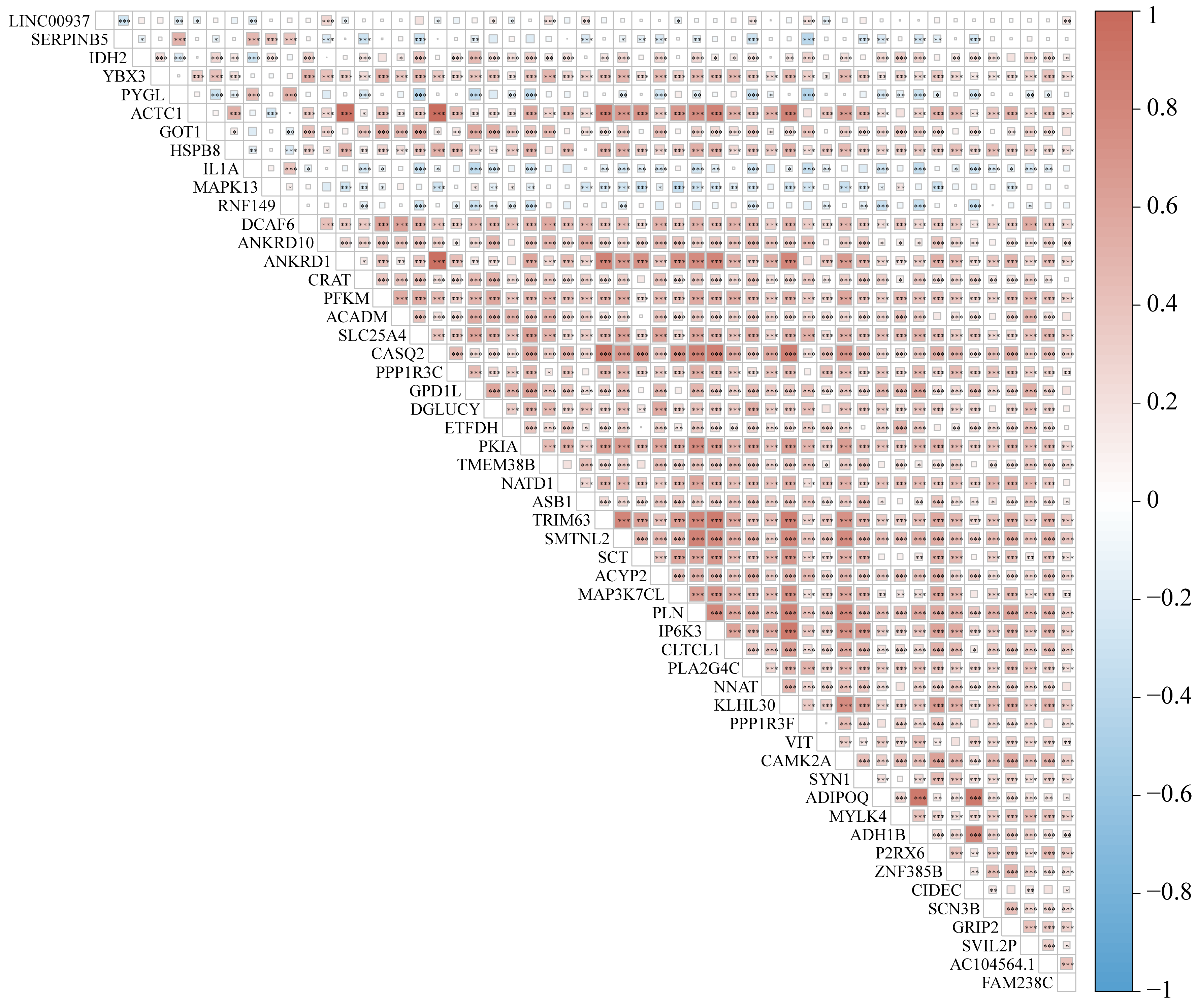

Supplement: Supplementary file 3 [file Image1.tif]
